# Twenty-five years of advocacy for patients with gastroparesis: support group therapy and patient reported outcome tool development

**DOI:** 10.1186/s12876-016-0523-3

**Published:** 2016-08-31

**Authors:** Teresa Cutts, Sandra Holmes, Archana Kedar, Karen Beatty, Mohammad K. Mohammad, Thomas Abell

**Affiliations:** 1 Division of Public Health Science, Department of Social Sciences and Health Policy, Wake Forest School of Medicine, Winston-Salem, USA; 2 Director of Nursing Education, Development and Research, King Saud Medical City, Riyadh, Saudi Arabia; 3 Division of Gastroenterology, Hepatology and Nutrition, Department of Medicine, University of Louisville, 550 South Jackson Street, ACB A3L15, Louisville, KY 40202 USA

**Keywords:** Gastroparesis, Patient-reported outcomes or PROs, Health-related quality of life, Support groups

## Abstract

**Background:**

Gastroparesis (Gp) is a poorly understood chronic gastrointestinal medical condition for which patient reported outcomes (PRO) are lacking. Previously developed symptoms scoring has been used for several decades. Using symptoms scores as a basis for documentation, 12 years of support/focus group patient feedback from the nearly 1000 attendees were integrated with medical care and recommendations for treatment were developed. Early attenders of the support group were compared with non-attendees for illness acuity, disability, and duration and number of office phone calls.

**Methods:**

Patients cared for in an academic medical practice were assessed for patient-derived PRO symptoms, coupled with standardized Health Related Quality of Life (HRQOL) measures. Based on factors identified by the patients via support/focus groups, a diagnostic and prognostic tool was developed.

**Results:**

The new tool utilized PRO symptoms and included provider assessments of medical illnesses as well as resource utilization. This ‘post PRO’ tool has been applied in a variety of settings for patients with the symptoms of Gp over the last two decades. The ‘pre-PRO’ factors from the support/focus groups were compared to the PRO measures as well as the ‘post-PRO’ scale to assess their usefulness. Using methods that combine chart data, including electronic medical records (EMR), with PRO symptoms may have design implications for PRO assessment. The resultant scales, as part of a new tool, can allow for sharing of PRO derived scores in a chronic gastrointestinal (GI), illness with different practitioners.

**Conclusions:**

These newly-derived scales offer a potentially useful tool for clinical decision-making, tailoring treatment to patient subgroups and engaging both patients and their families and caregivers in more active partnerships with providers to improve health outcomes.

**Electronic supplementary material:**

The online version of this article (doi:10.1186/s12876-016-0523-3) contains supplementary material, which is available to authorized users.

## Background

Gastroparesis (Gp) is a chronic disorder, which is classically defined as when the stomach takes too long to empty its contents; many patients have Gp symptoms even without measured delayed gastric emptying or gastroparesis-like syndrome, otherwise known as unexplained nausea and vomiting (UNAV) [1]. Together, these disorders can be called the Gp Syndromes. Common symptoms include chronic and/or recurrent vomiting, nausea, bloating/distension, loss of appetite/early satiety to meals and abdominal pain. The most common known causal agent of Gp is diabetes (Type I and Type II), accounting for 30 % of the cases [2]. Diabetic (DM) Gp or DM Gastropathy, if non delayed gastric emptying, is associated with high levels of blood glucose that cause chemical changes in nerves and blood vessels that supply oxygen and nutrients to nerves, especially the vagus nerve. Gp can occur with autonomic nervous system damage [3] as well as enteric nervous system damage, as a result of surgery on the stomach, and from viral infections. Roughly 70 % of people suffering from Gp have idiopathic gastroparesis, meaning the cause is unknown even after medical testing [4]. Complications from Gp include chronic nausea, bloating, vomiting, loss of appetite, weight loss and infection [3]. These complications can become so debilitating that many people suffering from Gp become bedridden and spend portions of their lives in and out of hospitals, greatly reducing the patients’ health related quality of life (HRQOL). Management by a team of clinicians skilled in addressing psychophysiological needs of the patient, facilitating patient and family education, and providing general support, can enhance coping and control, which positively impact quality of life [3, 5].

Over the course of 25 years, two of the authors (TA and TC) have worked with patients with symptoms of Gp. Consequently, they pursued the development of tools for patient experience reporting (which we term “pre-PRO or pre-Patient Reported Outcomes”), as a means of documenting the patient’s experience and making recommendations for advocacy and improved treatment of Gp. Such development of defined and reliable PROs for use in Gp patients has been recommended by the US Center for Drug Evaluation and Research (CDER) [6].

**Fig. 1:**
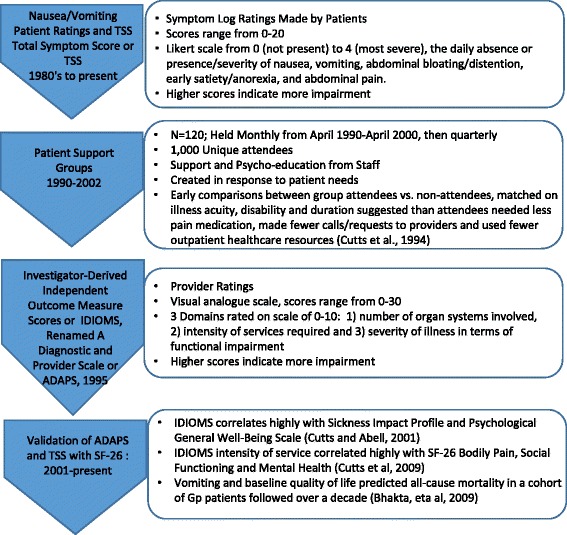
Process Flow of Development of Patient and Provider Rating Tools

This paper describes findings from 12 years of conducting supportive therapy groups with persons experiencing neuro-gastrointestinal disorders, particularly gastroparesis, measurement of health related quality of life, and the development of provider and patient rating tools (pre-PROs) in this group over two decades. Essentially, our earliest PRO tool development started in the 1980’s in our drug studies of patients treated with pharmacologic agents such as cisapride, with evaluation of nausea/vomiting parameters based on the metrics of FDA approved studies at the time [7]. These patient-reported outcome scales, which included measures of nausea, vomiting, anorexia/early satiety, bloating/distension and abdominal pain, on a 0–4 scale with none to worse, were also evaluated by their frequency and severity and can be summarized as a total symptom score (or TSS) by adding the symptoms together. This FDA derived PRO scale has been used by our patient population for the last 30 years. Based on learning from patients, and what the patients thought were their main concern from patient support and focus groups, we created the ADAPS/IDIOMS tools in 1995, which was used along with the PRO measures including TSS (Additional file 1). Finally, we conducted work further validating all these tools (patient symptoms by PRO/TSS with the tool of ADAPS/IDIOMS) and with the SF-36 in the 2000s (Additional file 2). Figure 1 gives highlights of this process and the tool development.

## Methods and approach

### Health Related Quality of Life (HRQL), Patient Reported Outcomes (PRO) with patient and provider rating tools

HRQL refers to either specific or global measures of subjective well-being. Tools for HRQL assessment can be useful in predicting outcomes, tailoring treatment and encouraging patient self-management of disease [8]. In addition to HRQL, patient-reported outcomes or PROs have become increasingly common in use for clinical care and tap similar domains as specific HRQL tools. PROs have been defined as “any report of the status of a patient’s health condition that comes directly from the patient, without interpretation of the patient’s response by a clinician or anyone else.”. These tools enable assessment of patient–reported health status for physical, mental and social well–being, a variant of an HRQL measure. A wide variety of patient-level instruments to measure PROs have been used for clinical research purposes. Many of these have been evaluated and cataloged within NIH’s Patient Reported Outcomes Measurement Information System (PROMIS). While PROMIS and other initiatives have validated patient-level outcome measures and instruments, there are two major challenges to using them for purposes of accountability and performance improvement:

1. They are not in widespread use in clinical practice.
2. Little is known about aggregating these patient-level outcomes for measuring the performance of the healthcare entity delivering care [9].

Early prototypes of PROs used by the authors, like patient symptom ratings, coupled with our provider rating tools, have aided in establishing a more holistic understanding of gastroparesis patients’ sense of well-being and the interruption of that sense of well-being due to complications related to gastroparesis. Particularly, our work has always included patient ratings (pre-PROs), as well as provider ratings. We contend that this mirror assessment offers a more comprehensive picture of the patient experience and provider view and lends itself to promoting both informed and activated patients and providers who feel more self-efficacy: the patient-provider partnership now advocated by the Institute for Healthcare Improvement and others [10]. Lastly, the development of defined and reliable PROs for use in Gp patients has been recommended by the CDER [6], who conclude that ideal PRO tools are lacking currently:

“Because gastroparesis is a symptomatic condition, a well-defined and reliable PRO instrument that measures all the clinically important signs and symptoms of gastroparesis would be the ideal primary efficacy assessment tool in clinical trials used to support labeling claims for the treatment of gastroparesis. However, at the current time, we know of no measure of clinically important gastroparesis signs and symptoms that would serve as the ideal primary efficacy assessment tool. Until an appropriate PRO instrument for gastroparesis becomes available, sponsors should consider the strategies discussed in the following sections when designing gastroparesis clinical trials. Sponsors may wish to explore new PRO instruments or novel diagnostic measures in early development, and potentially correlate the results with dose-ranging trials.”

We believe that the rating tools developed and described below constitute “new PRO instruments or novel diagnostic measures in early development” suggested by the CDER [6].

### Early drug studies and Pre-PRO tools

From early 1983 to present, one of the authors (TA) has served as a PI on studies exploring drugs and other treatments for gastroparesis, starting with Cisapride investigations in the mid-1980s. Early studies had the patients rate nausea and vomiting levels as part of structured clinical trials, a precursor to the Total Symptom Score or TSS tool described below [7, 11]. See Additional file 1 for an example of the utilized PRO/TSS.

### ADAPS/IDIOMS tools

Methodologies to determine the best patient care for people suffering from gastroparesis continued to be developed and refined by the corresponding author and his teams in the late 1980s through the 1990s. Along with the PRO/TSS, early work with patient ratings included a Symptoms Interview based on the severity and frequency of symptoms and the Short Form – 36 Version 1, a well-validated and psychometrically sound Health Related Quality of Life Measure. Two authors (TC and TA) then developed a provider-rating tool which was initially called A Diagnostic And Prognostic Score (or ADAPS) and later called the Investigator Derived Independent Outcomes Measure Score or IDIOMS. Essentially the ADAPS/IDIOMS tool was developed around 1995 to evaluate *three* specific areas of patient experience, based on what patients named as important to them: (1) what they wanted to be addressed medically; (2) how sick they were with GI symptoms other medical problems, and; (3) what services they needed, a gross healthcare utilization measure. These areas correspond to healthcare utilization domains used as components of the Diagnostic Related Groups (DRGs) that the Center for Medicare & Medicaid Services (CMS) uses to set reimbursement rates [12]. The GI specific provider rating or IDIOMS was also incorporated to determine its usefulness as a synergistic tool in cooperation with patient reported symptoms.

In the ADAPS/IDIOMS tool (Additional file 1) each of these three domains is assessed on a 1–5 scale and a total summed score, ranging from 1 to 30. Patient symptom ratings (termed total symptom scores or TSS), the IDIOMS and its earlier versions (e.g., the ADAPS) continually have been used by our GI motility team, in several locations, since 1995.

### Support group therapy/Focus groups

From April 1990 to April 2002, support group sessions were held for GI motility disorder patients at University of Tennessee Health Science Center. These sessions occurred monthly (except for 4 months), then staggered to quarterly for the last 2 years, for a total of about 120 sessions. Although detailed demographic records were not kept, over 1000 non-unique persons attended these groups over the 12-year period; attendance ranged from 5 to 45 patients plus additional GI academic and clinical staff per session. The group was started in part because the behavioral management strategies for GI symptoms used at that time (mostly to cope with musculoskeletal pain) did very little to aid GI motility disorder patients, and the team wished to offer other self-management strategies to help patients coping with these difficult illnesses. There were few medical options for GI motility patients; gastric electrical stimulation, now used in some drug refractory patients, was still investigational. Comparisons between early support group attendees and non-attendees, matched on illness acuity, disability and duration, suggested that attendees required fewer pain medication prescriptions, made fewer calls and requests of their providers, and used fewer outpatient healthcare resources, compared to non-attendees [8].

Support group format consisted of introductions (participants briefly telling their stories), followed by a professional speaker, and a subsequent question and answer period. Support group sessions lasted around 90 min, but often participants stayed for over 120 min. Focus group topics included pain management, partnering with your medical team, nutritional issues, total parenteral nutrition (TPN), family dynamics in chronic illness, navigating the insurance system, physical activity to improve motility function, coping with cyclic vomiting syndrome for adults and children, financial management when dealing with a chronic illness, autonomic nervous system function impact on GI symptoms, diabetes and GI motility disorder symptoms, coping with depression and anxiety, biofeedback for pain management, anger management, GI stimulation (pacing), dealing with social isolation secondary to nausea, vomiting, as well as explosive diarrhea and bloating.

Many patients found an understanding venue in which to share, often resulting in crying, anger or other strong negative emotions, but patients also laughed together. The group had many “inside” jokes and prided themselves on being able to laugh about vomiting and other socially unacceptable behaviors. Unilaterally, group members found that the group helped instill hope, as well as bolster their morale and courage in coping with Gp [8]. However, because the group was hospital-based and many of the more severely ill patients attended (with IV poles and in hospital gowns), some early outpatient attendees reported that seeing other patients with like illnesses who were so critically ill could be demoralizing. Some outpatients reported increased anxiety upon hearing about the rapid trajectory of illness severity and impairment for their hospitalized peers. Additionally, prior misdiagnoses and psychiatric overlay often placed upon patients (especially in rural areas with few specialists), generated many stories that shared how these experiences contributed to their pain and suffering. While all were allowed to share their stories and empathy was offered, the group focused on how to partner with healthcare professionals, versus feeling victimized by unintended iatrogenic impact from the medical and insurance systems.

The group also provided a forum for families and other patients to honor and memorialize those who died, and was a good venue for grief counseling for many families, once the family was ready to share about their loss. Unfortunately, many patients in our cohort (roughly 25 % from the initial University of Tennessee patient group) died in the course of those 12 years, with an 11.3 % mortality rate reported for a cohort of 214 who were treated with gastric electrical stimulation [13].

Additionally, the group often shared their complementary medicine modalities with one another and joked about writing a “GI Motility Handbook of Home Remedies.” Interesting home-based modalities included eating Vietnamese soup and greasy Krystal burgers or having a family member use a plunger to “suction” the patients’ back or side when bloating was painful, etc., all offering some measure of relief for some patients. The group facilitator did not promote these measures as alternative medicines or substitutes for allopathic medicine, and always urged patients to consult with the GI motility team.

These support groups provided a good venue for building team partnerships with families and patients to improve treatment and communication, before patient and family-centered care or activated patient concepts were considered important or even named [5]. Anecdotal information learned from patients about the experience of living with these disorders, as well as the impact on family life and coping were useful in creating/directing holistic treatment plans. Sharing from patients in these support groups helped the authors develop tools that combined health related quality of life measures or “pre-PRO” assessments, as ways to better capture and integrate the patient experience into treatment.

### Ethics, consent and permissions

All research and data analysis was approved by the University of Mississippi and University of Tennessee Institutional Review Boards and by IRBs at subsequent gastrointestinal (GI) team locations at the University of Mississippi and the University of Louisville. A waiver of consent was obtained from the authorizing IRB.

## Results

### Total symptom score or TSS

Our team initially reported that prospective ratings of vomiting and baseline quality of life were good predictors of all-cause mortality in Gp patients followed for several years [14]. We then explored the association between baseline patient-generated symptoms of nausea and vomiting frequency and severity as a PRO tool with SF-36 scales in patients with Gp and reported on the use of patient nausea/vomiting ratings including the TSS as a PRO tool in 2009 [15]. Patient symptom ratings of frequency and severity of nausea, vomiting and epigastric pain were examined at baseline in a large cohort of patients with Gp. The SF-36, a well-standardized functional measure of subjective well-being and HRQOL, has been shown to be useful in demonstrating the impact of illness in various patients with various GI disorders, particularly Gp. We compared baseline SF-36 scores and nausea and vomiting ratings in 235 patients with gastroparesis, mean age 47 years; 79 % female, 21 % male; 82 % Caucasian, 17 % African-American, 1 % other ethnicity. Etiology of Gp for those rated was idiopathic (53 %), diabetic (29 %) and post-surgical (12 %). Significant Pearson correlation coefficients were found between SF-36 Social Functioning and vomiting severity (*r* = −.236, *p* = .0001). SF-36 Role Emotional correlated significantly with epigastric pain severity (*r* = .194, *p* = .003).

**Fig. 2:**
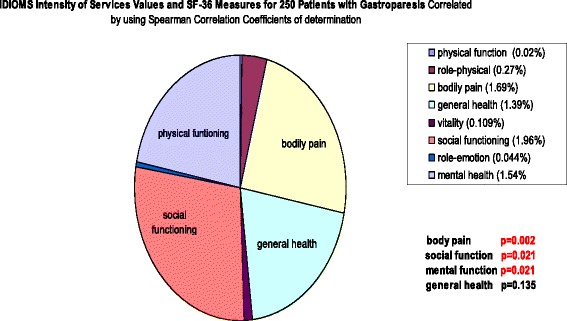
IDIOMS Tool and Correlations with SF-36 Measure in a Sample of Patients with the Symptoms of Gastroparesis

**Fig. 3:**
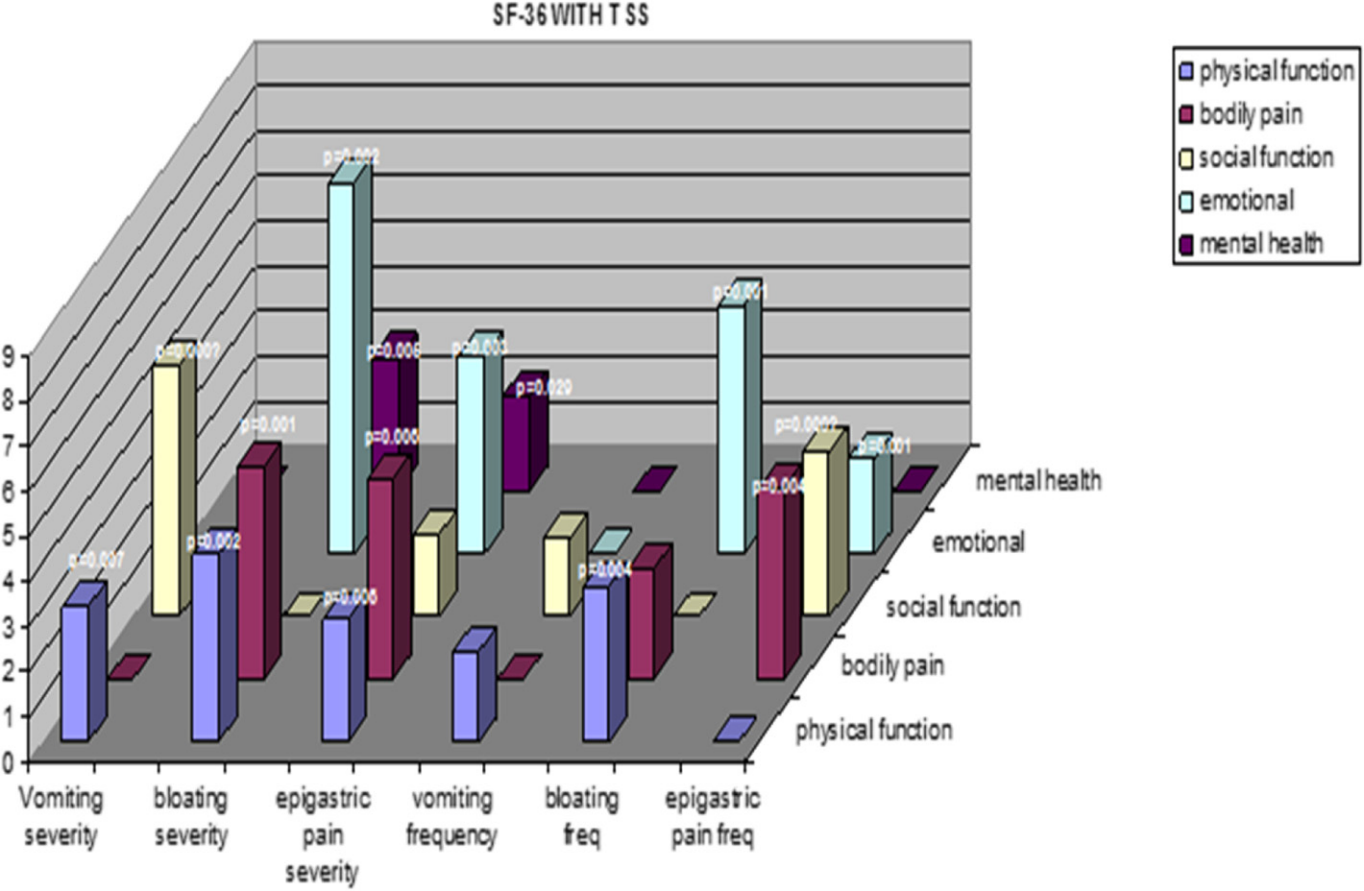
Correlation of Patient PROs Reported as GI Total Symptom Scores with SF-36 Measures in a Sample of Patients with the Symptoms of Gastroparesis

**Fig. 4:**
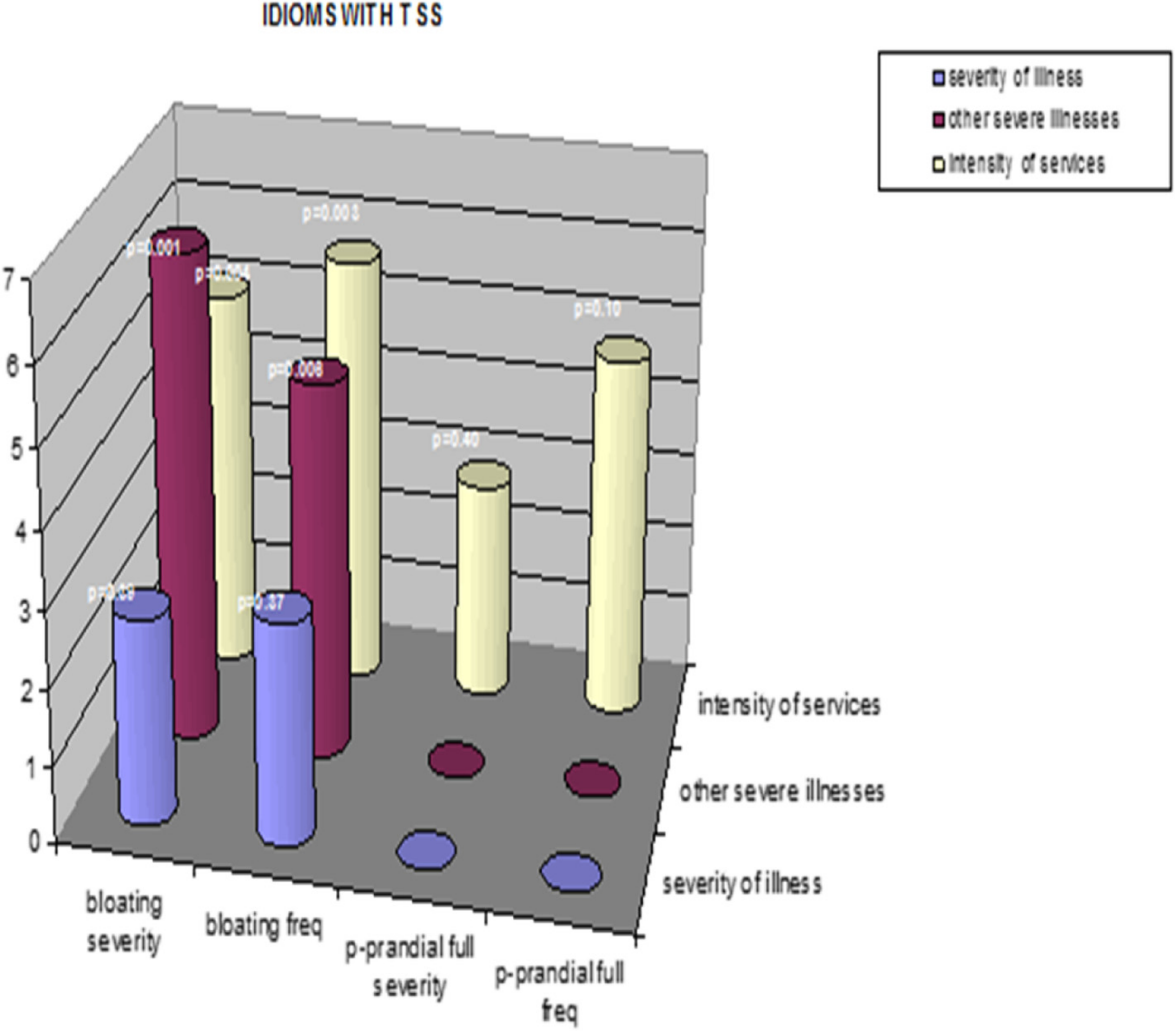
Correlations of IDIOMS Tool with patient PRO Measures Reported as GI Total Symptom Scores in Patients with the Symptoms of Gastroparesis

Examining the same cohort of patients with Gp, we reported more in-depth correlations on the association between baseline patient-generated symptom logs with SF-36 scales [16]. Symptom logs included the Total Symptom Score (TSS), ratings made by patients, rating frequency and severity of nausea, vomiting, bloating, early satiety, epigastric pain and burn, postprandial fullness and cardiac burn. Significant Pearson correlation coefficients, both positive and negative, were found between SF-36 Physical Functioning score and bloating severity (*r* = −.202, *p* = .002) and bloating frequency (*r* = −.188, *p* = 004) and epigastric pain (*r* = −.180, *p* = .006). SF-36 Bodily Pain scores correlated significantly with bloating severity (*r* = − .220, *p* = 001), bloating frequency (*r* = − .186, *p* = .005), epigastric pain severity (*r* = −.190, *p* = .006) and epigastric pain frequency (*r* = −.196, *p* = .004) and epigastric burn frequency (*r* = −.204, *p* = .003). SF-36 Social Functioning also correlated strongly with epigastric pain frequency (*r* = −.188, *p* = .005) and vomiting severity (*r* = −.236, *p* = .000). SF-36 Role Emotional correlated significantly with bloating severity (*r* = −.284, *p* = .000) and bloating frequency (*r* = −.225, *p* = .001) and epigastric pain severity (r=. 194, *p* = .003). Lastly, SF-36 mental health scores correlated significantly with bloating severity (*r* = −.182, *p* = .005) (Figs. 2, 3 and 4).

PROs via our GI symptom ratings of vomiting frequency and severity, as well as epigastric pain severity, demonstrated strong associations with SF-36 measures of Social and Emotional functioning level, in the baseline ratings of this Gp cohort. In this sample, vomiting and pain have a strong impact on patients’ subjective ratings of well-being. PROs via our GI symptom ratings of bloating frequency and severity, as well as epigastric burn and pain severity and frequency, demonstrated strong associations in the baseline ratings of this GP cohort as well. These findings suggest that these GI symptom ratings possess some degree of concurrent validity with sub-scales of the SF-36 and could be used as PROs.

### Validation of IDIOMS and TSS with HRQOL measures

Finally, we reported on the concurrent validity and use of our PRO symptoms and, the Investigator-Derived Independent Outcome Measures Scores (IDIOMS) in this cohort of patients with Gp [17]. Early studies demonstrated that the IDIOMS correlated highly with some global measures of HRQOL measures, including the Sickness Impact Profile and Psychological General Well-Being Scale. When we compared baseline IDIOMS and SF-36 scores in 242 patients with Gp, significant Spearman correlation coefficients were found between IDIOMS intensity of service and SF-36 Bodily Pain (*p* = .002), Social Functioning (*p* = .021) and Mental Health (*p* = .021). These findings suggest that the IDIOMS does possess concurrent validity with sub-scales of the SF-36, and merits further investigation as such a tool to measure treatment outcomes. Highlights of these findings are presented in Figs. 2, 3 and 4.

The IDIOMS tool has been demonstrated to have high inter-rater reliability (*r* = 0.96, *p* < 0.001) in a study of 134 Gp patients rated at multiple intervals by two independent practitioners [18]. Convergent validity, or how well a measure correlates with standardized and psychometrically proven HRQOL measures of the same domains, constructs or areas, has also been demonstrated for the IDIOMS with the Sickness Impact Profile (SIP) and the Psychological General Well-being Scale (PGWB), as well as patient symptom ratings, in earlier studies of intractable Gp patients, some undergoing gastric electrical stimulation (GES).

Likewise, the IDIOMS tool has shown to have good predictive validity, or clinical responsiveness, which is the ability of an instrument to demonstrate a difference in measurement that corresponds with symptom change from baseline after treatment or in predicting outcome. Several studies have demonstrated that the ADAPS/IDIOMS is clinically responsive in short-term treatment of GES, in a longer term follow-up of GES patients, and in patients treated with both prokinetics and biofeedback [19]. A recent study of 441 consecutive Gp patients showed that the baseline IDIOMS score was a highly significant indicator of death (*p* <0.001), as was the symptom rating of vomiting at baseline (*p* = 0.0333) [10].

In a recent cohort of Gp patients, bloating and abdominal pain were commonly reported as the initial onset symptoms perceived by Gp patients progressing with severity of the disease to nausea and vomiting. PRO-reported bloating and abdominal pain severity scores significantly correlated with abnormal gastric motility measures indicative of a progressive disease state [20]. In this cohort of gastroparesis patients (with mean symptom duration just over 7 years), we concluded that ADAPS/IDIOMS shows usefulness in terms of serving as a brief and reliable PRO tool, mainly in terms of prognosis. Given that it has now been shown to have some convergent validity in terms of correlating with specific subscales of the SIP, Psychological General Well-Being Scale [17], and the SF-36 (particularly Role-Emotional and Bodily Pain), we think that the IDIOMS is promising as a PRO tool to supplement more invasive and longer global measures of HRQOL.

## Discussion

The use of patient-reported outcomes has become increasingly important in all aspects of medical care. The FDA recently reviewed the application of PRO measures related to studies for patients with gastroparesis [6]. As pointed out in this document, newer measures of patients’ symptoms, although widely studied, have not been shown to be superior to the PRO/TSS measures used in this report.

Although not originally designed for use in patient-centered disease-management efforts, our team believes that incorporating the IDIOMS tool into clinical care could be useful as a complementary healthcare utilization measure. These sorts of tools, coupled with traditional PRO measures including TSS, could be used in improving patient symptoms and outcomes, particularly in a primary care setting, and could potentially be used in other patient-provider interactions, such as Gp self-management training (e.g., similar to Stanford’s Chronic Care Management community-based trainings). Additionally, as suggested earlier, the IDIOMS tool offers a clinician-friendly measure that could be used with a variety of chronic conditions, beyond Gp, as a population health management tool [3].

Future comparative or concurrent validity studies with the IDIOMS tool with more widely-used GCSI and PAGI-QOL tools, could be useful in discerning exactly what instruments are best suited for patient needs, PRO protocol development and population health management. For example, such an assessment tool package could shape and form more tailored treatment for patients based on etiology and severity of motility disorder conditions, as well as other chronic co-morbid conditions. Additional work with the IDIOMS tool can include further use for stratification of patient sub-types, for both clinical and clinical research applications, and the correlation of IDIOMS with subsequent health care costs when following patients longitudinally.

## Conclusions and recommendations

One of the difficulties in using global HRQOL tools is that they fail to comprehensively reflect the holistic experience of gastroparesis patients having a traumatic illness that greatly impairs quality of life over several years [21]. Generic HRQOL measures do not adequately reflect severity of illness, extent of other organ involvement (which is often extensive in patients with Gp), or utilization of healthcare resources. Additionally, recent European studies suggest that only 40 % of patients actually complete PROs as requested by PCPs or other providers [22]. Our IDIOMS tool may fill a significant gap in reflecting the full clinical experience of the illness, which is a primary goal of patient-reported outcomes. Further study of our tool is needed with more patients, at earlier stages of their Gp course, to validate and refine its clinical utility and effectiveness across the spectrum, from those with early diagnoses and more minimal symptoms to those with intractable symptoms. It is likely that early detection could be improved by educating and targeting primary care providers who often are the first practitioners to encounter patients manifesting the early symptoms of Gp.

Patient reports supported the clinical utility of the IDIOMS and PRO/TSS tools from an HRQL standpoint. In anecdotal reports gleaned from our support groups, patients reported that learning behavioral strategies was helpful in improving HRQOL by enhancing overall coping skills and decreasing their sense of hopelessness/helplessness. Such strategies included limiting known nutritional substances that exacerbate bloating, pain coping or energy pacing techniques and managing anxiety at the beginning of a vomiting cycle.

In summary, the IDIOMS and PRO/TSS tools offer both a brief clinician-friendly instrument that has shown good inter-rater reliability and concurrent validity with a series of different global standardized measure subscales of HRQOL (e.g., SIP, PGWB, SF-36), as well as a patient rating that has been used extensively. The SF-36 Role-Emotional and Bodily Pain scales are related to the IDIOMS Intensity of Service and Other Organ Involvement scales. The IDIOMS Intensity of Service scale is also correlated with bloating severity and frequency [17]. We also suspect that the IDIOMS, which is based on patient self-reports, may be a useful complement to other PRO measures in any chronic GI syndrome (e.g., IBS or functional dyspepsia), to reflect the holistic experience of these illnesses over time. Additionally, incorporating the IDIOMS and TSS tools as patient-related outcome measures can help providers understand the phenomenological experience of the illness itself, a most important variable in promoting patient self-management efforts in these chronic and often debilitating illnesses [21]. As such, we believe these tools deserve further study with Gp cohorts, to continue to refine their usefulness in early assessment and possible tailoring of interventions for different stages of the disease process in Gp and other chronic GI syndromes.

Lastly, The National Quality Forum, in its December 2012 report, advocated for use of PROs as part of a population health management strategy [3]. We propose that our IDIOMS and PRO/TSS tools could be part of an ensemble of measures that could be used, not only for chronic GI illnesses like gastroparesis, but also for a host of other chronic conditions, such as diabetes, obesity, cardiovascular disease and others.

## Additional files

Additional file 1: PRO/TSS Tool. Total Symptom Score instrument used as a Patient Reported Outcomes tool. (DOCX 11 kb) Additional file 2: ADAPS/IDIOMS Tool. A Diagnostic and Predictive Score/Investigator Derived Independent Outcome Measure Score Tool. (DOC 25 kb)

## Abbreviations

ADAPS: A diagnostic and prognostic score
CMS: Center for medicare & medicaid services
DRG: Diagnostic related groups
EMR: Electronic medical records
GES: Gastric electrical stimulation
GI: Gastrointestinal
Gp: Gastroparesis
HRQOL: Health related quality of life
IDIOMS: Investigator derived independent outcomes measure score
IOS: Intensity of services
OSI: Other significant illnesses
PAGI: Patient assessment of gastrointestinal disorders
PRO: Patient-reported outcomes
PROMIS: Patient reported outcome measurement information system
SF-36: Short form-36
SOI: Severity of illness
TPN: Total parenteral nutrition
TSS: Total symptom score
