# Supplementary material for: Twenty-five years of advocacy for patients with gastroparesis: support group therapy and patient reported outcome tool development
Source: BMC Gastroenterol. 2016 Aug 31;16(1):107. doi: 10.1186/s12876-016-0523-3 (PMC5006521; doi:10.1186/s12876-016-0523-3)
Supplement: Additional file 1: — PRO/TSS Tool. Total Symptom Score instrument used as a Patient Reported Outcomes tool. (DOCX 11 kb) [file 12876_2016_523_MOESM1_ESM.docx]

Patient Reported GI Outcomes and Total Symptom Score

Rated as 0-4, None to Worse and Total (0-20)

Symptom Frequency Severity Average

Vomiting 0 1 2 3 4 0 1 2 3 4 0 1 2 3 4

Nausea 0 1 2 3 4 0 1 2 3 4 0 1 2 3 4

Anorexia/Early Satiety 0 1 2 3 4 0 1 2 3 4 0 1 2 3 4

Bloating/ Distension 0 1 2 3 4 0 1 2 3 4 0 1 2 3 4

Abdominal pain 0 1 2 3 4 0 1 2 3 4 0 1 2 3 4

Total GI Symptoms score (Sum of above) (Sum of above) (Sum of above)
