# Supplementary material for: Twenty-five years of advocacy for patients with gastroparesis: support group therapy and patient reported outcome tool development
Source: BMC Gastroenterol. 2016 Aug 31;16(1):107. doi: 10.1186/s12876-016-0523-3 (PMC5006521; doi:10.1186/s12876-016-0523-3)
Supplement: Additional file 2: — ADAPS/IDIOMS Tool. A Diagnostic and Predictive Score/Investigator Derived Independent Outcome Measure Score Tool. (DOC 25 kb) [file 12876_2016_523_MOESM2_ESM.doc]

**INVESTIGATOR DERIVED INDEPENDENT OUTCOME MEASURE SCORES (IDIOMS)**

**A DIAGNOSTIC AND PREDICTIVE MODEL (ADAPS)**

**TOTAL SCORES = INDEPENDENT DIAGNOSTIC OUTCOME MEASURE**

PATIENT NAME: DATE: BY:

_____________________________________________________________________________________

___1 2 3 4 5 6 7 8 9_ 10

NONE MILD MODERATE SEVERE VERY SEVERE

1. SEVERITY OF ILLNESS (SOI)

NONE MILD MODERATE SEVERE VERY SEVERE

SYMPTOMS SYMPTOMS SYMPTOMS SYMPTOMS

B. ORGANS INVOLVED SYSTEMS (OSI)

One GASTROINESTINAL Two ORGANS Three ORGANS > 3 ORGANS

ORGAN

C. INTENSITY OF SERVICES (ISO)

MEDICINES MEDICINES AND MEDICINES, HOME MEDICINES,

HOME HEALTH HEALTH, HOSPITILI- HOME HEALTH

ZATIONS MULTIPLE HOSPITALI-

ZATIONS

Adapted from Cutts, et al, NGM, 2005
